# Rituximab maintenance improves overall survival in follicular lymphoma: A retrospective nationwide real‐world analysis from Taiwan Cancer Registry Database

**DOI:** 10.1002/cam4.1622

**Published:** 2018-07-15

**Authors:** Huai‐Hsuan Huang, Yao‐Chun Wen, Ho‐Min Chen, Fei‐Yuan Hsiao, Bor‐Sheng Ko

**Affiliations:** ^1^ Division of Hematology Department of Internal Medicine National Taiwan University Hospital Taipei Taiwan; ^2^ Health Data Research Center National Taiwan University Taipei Taiwan; ^3^ Graduate Institute of Clinical Pharmacy National Taiwan University Taipei Taiwan; ^4^ School of Pharmacy National Taiwan University Taipei Taiwan; ^5^ Department of Pharmacy National Taiwan University Hospital Taipei Taiwan

**Keywords:** follicular lymphoma, nationwide study, overall survival, rituximab maintenance, Taiwan Cancer Registry Database

## Abstract

Follicular lymphoma (FL) is the most frequent indolent lymphoma in Western countries, but it is less frequent in Asia. Several trials have demonstrated the progression‐free benefit of rituximab maintenance for FL patients in Western countries. However, the overall survival (OS) benefits and effectiveness of rituximab maintenance in Asian FL patients remain uncertain. We utilized the Taiwan Cancer Registry Database and the National Health Insurance Research Database to investigate the roles of rituximab maintenance for newly diagnosed FL patients in Taiwan. Among 836 patients with newly diagnosed FL during 2009‐2012, we enrolled patients with stage II‐IV diseases receiving 4‐8 cycles of rituximab‐containing induction chemotherapies (R‐induction). We excluded those who died or received additional chemotherapy within 180 days after R‐induction. Among the 396 enrolled patients, 260 underwent rituximab maintenance (R‐maintenance group), and 136 served as the observation group. The R‐maintenance group received less anthracycline and fewer cycles of R‐induction than the observation group, but they exhibited a significantly better OS both in the univariate and multivariate analyses [hazard ratio, 0.42; 95% confidence interval, 0.19‐0.91] after adjusting for age, sex, and Ann Arbor stages. Meanwhile, we also found more patients required further therapies in the first 6 months after the cease of rituximab maintenance. In the subgroup analysis, patients older than 60 years or with stage IV diseases benefited more from rituximab maintenance. Conclusively, our nationwide study is the first one to demonstrate the OS benefit of rituximab maintenance after induction therapies in newly diagnosed FL patients from Asian populations.

## 1 INTRODUCTION

Follicular lymphoma (FL) is the most frequent type of indolent B‐cell lymphoma (BCL) in Western countries.^1^ In contrast, the incidence is relatively lower in Asian populations.^2^ Patients with localized and early‐stage FL are treated aggressively with radiotherapy at the involved sites.^3^, ^4^ Additional chemoimmunotherapy only improves progression‐free survival instead of overall survival for the patients with early‐stage FL.^3^ On the contrast, patients with asymptomatic advanced‐stage FL do not need to be treated immediately.^1^, ^5^, ^6^, ^7^ Once they develop symptoms, chemotherapies with rituximab are suggested as frontline treatments.^1^, ^4^, ^8^, ^9^, ^10^, ^11^

Rituximab is a chimeric monoclonal antibody against CD20, a common B‐cell surface marker in BCL, including FL. Rituximab combined with cytotoxic chemotherapies is superior to chemotherapies alone, regardless of frontline treatments or in relapse and/or refractory FL.^8^, ^9^, ^10^, ^12^, ^13^, ^14^, ^15^ Several prospective trials tried to address the clinical benefits of rituximab maintenance in the patients with FL, but rituximab maintenance only improves progression‐free survival instead of overall survival.^16^, ^17^, ^18^, ^19^ The clinical benefit of rituximab maintenance is still uncertain. The PRIMA study was a phase III randomized study performed to compare the benefit of rituximab maintenance in FL patients previously treated with frontline immune‐chemotherapy.^16^ Although the study revealed longer progression‐free survival (PFS) in patients undergoing rituximab maintenance, no advantage for overall survival (OS) was demonstrated.^16^ Other studies also demonstrated only a progression‐free benefit of rituximab maintenance after re‐induction therapies in relapse FL.^17^, ^18^ Currently, no prospective randomized study has demonstrated the overall survival benefit of rituximab maintenance in the patients with FL. Only one meta‐analysis and one retrospective analysis showed the overall survival benefit of rituximab maintenance, and the most of included patients lived in the Western countries.^15^, ^20^ For Asian FL patients, only one phase II single‐arm Japanese study demonstrated the safety of rituximab maintenance in indolent BCL with high tumor burden.^21^ Currently, there is only limited data to support the long‐term survival improvements of rituximab maintenance, especially for Asian patients with FL.

The Taiwan Cancer Registry Database (TCRD) has been initiated from 1979.^22^ The hospitals with a capacity of >50 beds must participate in the TCRD. In 2012, the completeness of the TCRD was 98.4%, and 91.5% of the incident cases had histological or cytological verifications for the diagnosis of cancers.^22^, ^23^, ^24^ In addition, the National Health Insurance (NHI) system in Taiwan is a mandatory and single‐payer health insurance system, which has been operated by the government from 1995. This insurance system covers >99% of residents in Taiwan. The NHI Research Database (NHIRD) collects all clinical information from the NHI and provides these data for further analyses. On the other hand, rituximab has been approved by NHI for the frontline treatments from 2006 and for 2‐year maintenance from 2008 for FL patients in Taiwan. Therefore, it makes possible for us to combine the 2 databases from the TCRD and the NHIRD to study the real‐world utilization of rituximab in Taiwan.

In our study, we incorporated the clinical information from the TCRD with the NHIRD to investigate the real‐world benefit of rituximab maintenance in Taiwanese FL patients. We identified the patients who had complete response after rituximab‐containing induction chemotherapies and stratified the patients into 2 groups according to the use of rituximab maintenance. Further, we compared the effects of rituximab maintenance in the patients with FL. To the best of our knowledge, our study is the first study to illustrate the real‐world benefits of rituximab maintenance in the Asian FL patients.

## 2 METHODS

### 2.1 Data sources

We used the clinical information from the TCRD between 2009 and 2013 and linked it with the NHIRD and the National Death Registry Database (NDRD) in Taiwan.^25^ The TCRD provides cancer‐specific information, including primary cancer site, date of diagnosis, histological type, and cancer stages.^22^ The NHIRD, which is maintained by the Health and Welfare Data Science Center (HWDC) of the Ministry of Health and Welfare, complements the TCRD data with claim‐based information on demographics, clinical, medical resource utilization (including outpatient and inpatient visits), costs of services, and treatment patterns. The NDRD was also used to ascertain the survival status for the study population. All databases were linked by scrambled patient identification numbers and analyzed in the HWDC to protect patient confidentiality.

### 2.2 Ethics statement

The study protocol was approved by the Research Ethics Committee of the National Taiwan University Hospital (NTUH‐REC‐201604051W).

### 2.3 Study design and study cohort

**Figure 1:**
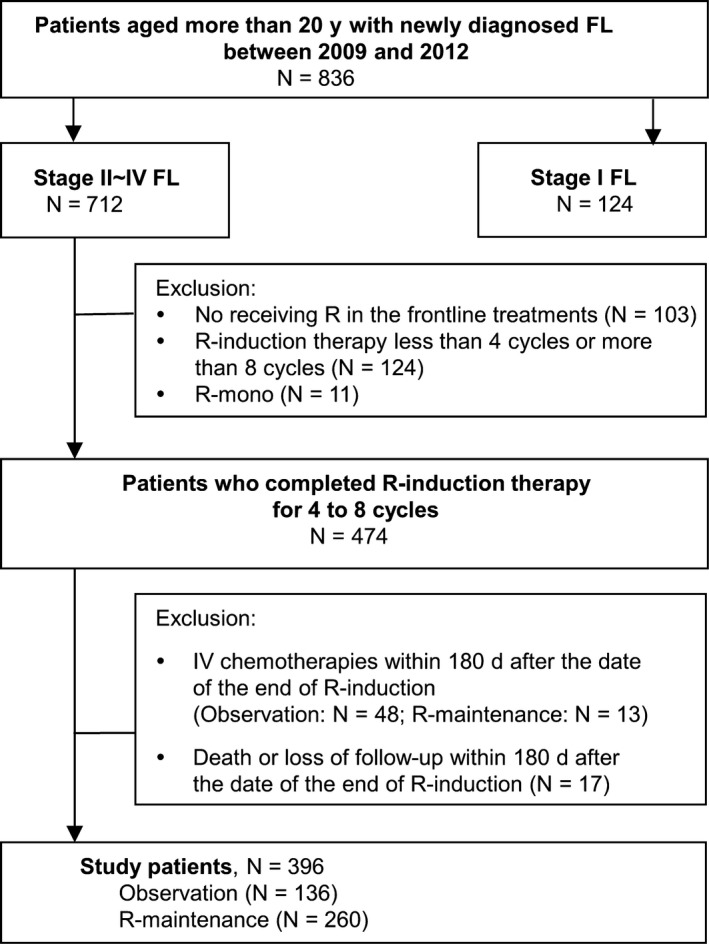
Algorithm of study cohort selection. FL, follicular lymphoma; R, rituximab; R‐induction, rituximab‐containing induction chemotherapy; R‐mono, rituximab monotherapy; R‐maintenance, rituximab maintenance

Eight hundred and thirty‐six adult patients were identified from the TCRD, and they were older than 20 years with newly diagnosed FL between 2009 and 2012. First, we excluded 124 patients with stage I disease (Figure 1). Second, we also excluded patients who did not receive rituximab after diagnosis (n = 103), patients undergoing <4 (n = 122) or >8 cycles (n = 2) of rituximab‐containing induction chemotherapies (R‐induction), or patients exclusively receiving rituximab monotherapy as the induction therapy (n = 11, R‐mono; Figure 1). Therefore, we only included patients with stage II‐IV FL and receiving 4‐8 cycles of R‐induction for further analysis (n = 474; Figure 1).

Because limitations were expected in assessing the responses of R‐induction in our database, we excluded the patients who underwent additional intravenous chemotherapies or died within 180 days after the end date of R‐induction (n = 78; Figure 1), which indicated disease progression or poor response for R‐induction. Three hundred and ninety‐six eligible patients were further stratified into 2 groups according to the administration of rituximab maintenance. If the patients received rituximab maintenance within 180 days after the end of R‐induction, they were defined as the R‐maintenance group (n = 260; Figure 1). The others were defined as the observation group (n = 136; Figure 1). The date of the 180th day after the last R‐induction chemotherapy was defined as the index date for our survival analysis.

### 2.4 Outcomes of interest

The outcomes of interest in this study included OS and time to treatment failure (TTF). OS was defined as the duration between the index date and death or the end of 2014, whichever occurred first. Patients alive in the end of 2014 were censored. TTF was defined as the duration between the index date and the date of the initiation of additional intravenous chemotherapies, radiotherapies, death, or the end of 2014, whichever occurred first. Patients would be censored if they were still alive and did not receive any additional intravenous chemotherapy or radiotherapy in the end of 2014. If the interval between 2 doses of rituximab was more than 6 months, we defined the patients with relapse and receiving rituximab monotherapy as the salvage therapy. The maximal cycle payed by the NHI in Taiwan was 8 cycles. The ninth cycle of rituximab monotherapy was also defined as the salvage therapy for relapse.

### 2.5 Statistical analyses

Continuous variables are presented as the mean with standard deviation (SD) or the median with first quartile (Q1) and third quartile (Q3). Categorical variables are presented as numbers and percentages. The significance of the differences in the baseline characteristics between 2 groups was assessed by Student's *t* test for continuous variables and chi‐square test for categorical variables. Kaplan‐Meier survival curves with log‐rank tests were used to examine the differences between 2 groups for analysis of OS or TTF. Univariate and multivariate Cox proportional hazard models were used to estimate the hazard ratios (HRs) and 95% confidence intervals (95% CIs) for OS and TTF, considering the treatment groups as independent variables. We further included sex, age groups, Ann Arbor stage, practice setting, the treatment types and cycles of R‐induction, and Charlson comorbidity index (CCI) as covariates in the adjusted model. Preplanned subgroup analyses were conducted to evaluate whether the effects of rituximab maintenance were consistent across different patient groups. The factors for subgroup analysis included age (between 20 and 59 years vs >60 years), Ann Arbor stages (stage II and III vs stage IV), induction treatment types (R‐CHOP vs R‐others), and the cycles of R‐induction (4‐6 cycles vs 7‐8 cycles). All of the analyses were performed using SAS, version 9.4 (SAS Institute Inc., Cary, NC, USA).

## 3 RESULTS

### 3.1 Patients with rituximab maintenance tended to receive less anthracycline and less cycles in rituximab‐containing induction chemotherapies

We identified 396 FL patients who fulfilled all the eligibility criteria as our study cohort (Figure 1). Among them, 260 (65.7%) received rituximab maintenance (the R‐maintenance group), and the other 136 (34.3%) served as the observation group. The baseline characteristics of the R‐maintenance and observation groups were presented in Table 1. The distributions of age, sex, Ann Arbor stages, practice settings, and CCIs were similar between 2 groups. Patients in the R‐maintenance group received less anthracycline (less R‐CHOP, *P*‐value .0150) and fewer cycles (*P*‐value .0010) in induction chemotherapies. Because the patients in the R‐maintenance group received less cycles of induction chemotherapies, their cumulative dose of rituximab was lower than that of the patients in the observation group (mean ± SD, 3634.6 ± 748.0 mg vs 3904.4 ± 941.9 mg, respectively; *P*‐value .0020).

**Table 1:** Comparison of baseline characteristics between patients undergoing rituximab maintenance (R‐maintenance) or observation

Variable	Category		All patients	R‐maintenance	Observation	*P*‐value
Patient number		n (%)	396 (100.0)	260 (100.0)	136 (100.0)	
Gender	Male	n (%)	196 (49.5)	130 (50.0)	66 (48.5)	.7811
	Female		200 (50.5)	130 (50.0)	70 (51.5)	
Age (years)		Mean ± SD	56.3 ± 12.3	55.8 ± 12.1	57.21 ± 12.71	.2654
Ann Arbor stage	II‐III	n (%)	219 (55.3)	142 (54.6)	77 (56.6)	.7035
	IV		177 (44.7)	118 (45.4)	59 (43.4)	
Practice setting	Medical center	n (%)	286 (72.2)	187 (71.9)	99 (72.8)	.8542
	Others		110 (27.8)	73 (28.1)	37 (27.2)	
Charlson comorbidity index	0	n (%)	257 (64.9)	168 (64.6)	89 (65.4)	.5958
	1		85 (21.5)	59 (22.7)	26 (19.1)	
	2+		54 (13.6)	33 (12.7)	21 (15.4)	
Time from diagnosis to R‐induction treatment (day)		Mean ± SD	73.2 ± 127.9	73.4 ± 125.3	72.9 ± 133.4	.0865
Induction treatments	R‐CHOP	n (%)	229 (57.8)	139 (53.5)	90 (66.2)	.0150^e^
	R‐others^b^		167 (42.2)	121 (46.5)	46 (33.8)	
Rituximab cycles in induction treatment	4‐6 cycles	n (%)	272 (68.7)	193 (74.2)	79 (58.1)	.0010^e^
	7‐8 cycles		124 (31.3)	67 (25.8)	57 (41.9)	
Relapse^a^		n (%)	123 (31.0)	83 (31.9)	40 (29.4)	.6081
Treatments after relapse	R	n (%)	62 (15.7)	48 (18.5)	14 (10.3)	.0199^e^
	R + CT^c^	n (%)	29 (7.3)	14 (5.4)	15 (11.0)	
	CHOP	n (%)	12 (3.0)	6 (2.3)	6 (4.4)	
	Others^d^	n (%)	20 (5.1)	15 (3.8)	5 (3.7)	
HSCT		n (%)	8 (2.0)	5 (1.9)	3 (2.2)	

CHOP, cyclophosphamide, anthracycline, vincristine, and steroid; CT, chemotherapies; HSCT, hematopoietic stem cell transplantation; R, rituximab; R‐induction, rituximab‐containing induction chemotherapies; R‐others, rituximab with chemotherapies other than CHOP; SD, standard deviation.

a Patients who received another intravenous therapies during following up were defined as relapse.

b In the R‐other group, 160 patients received R‐CVP and 7 patients received other rituximab‐containing chemotherapies than R‐CHOP and R‐CVP.

c Six patients received R‐CVP, 4 patients received R‐CHOP, 5 patients received fludarabine‐based chemotherapies with rituximab, and 14 patients received rituximab with oral chemotherapies.

d Others included nonrituximab‐containing chemotherapies other than CHOP, such as oral chemotherapies, CVP, and fludarabine‐based chemotherapies.

e *P*‐value is <.05.

With a median follow‐up of 2.6 years, 12 (4.6%) and 15 (11.0%) patients died among the R‐maintenance and observation groups, respectively. In addition, 83 (31.9%) and 40 (29.4%) patients experienced relapses and initiated additional intravenous rituximab or chemoimmunotherapies among the R‐maintenance and observation groups, respectively (Table 1). After relapse, most of the patients in the R‐maintenance group received rituximab only; in contrast, most of the patients in the observation group received rituximab‐containing chemotherapy (Table 1). Eight patients underwent hematopoietic stem cell transplantations later (Table 1).

### 3.2 Rituximab maintenance prolonged overall survival for the patients who had good response after rituximab‐containing induction chemotherapies

**Figure 2:**
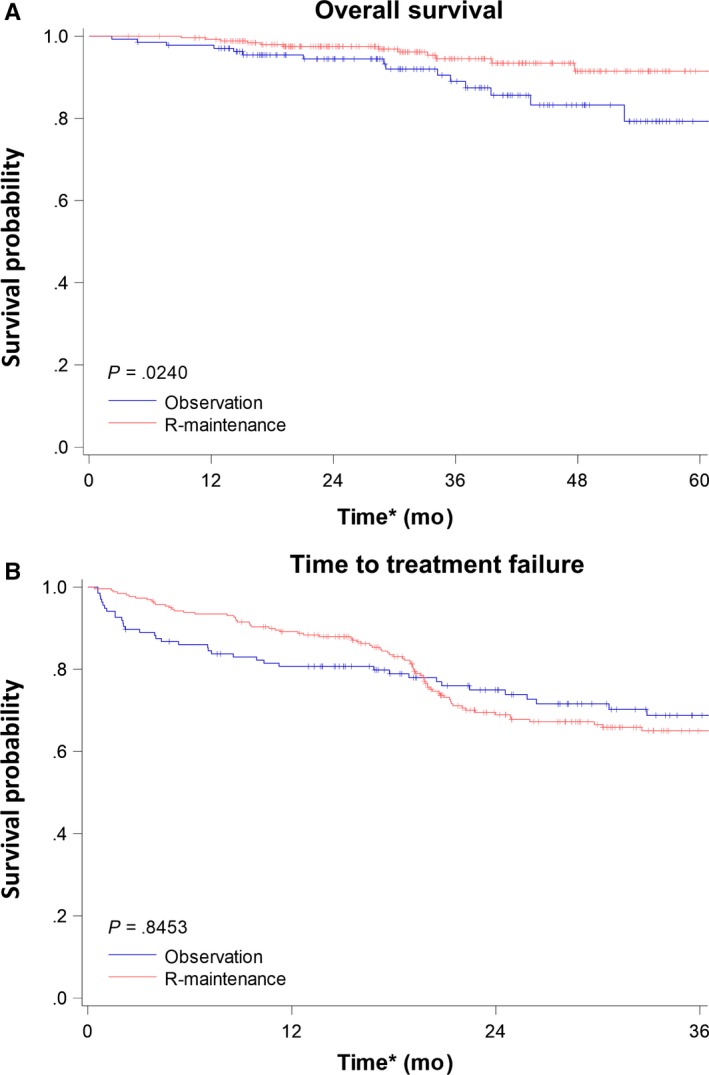
Kaplan‐Meier plots of overall survival (OS) and time to treatment failure (TTF) for enrolled patients. A, OS: Patients in the R‐maintenance group had better overall survival compared with those in the observation group. B, TTF: There was no statistical difference between the 2 groups. *The index date (Day 0) was the 180th day after the end date of the last rituximab‐containing induction chemotherapies

Kaplan‐Meier survival analysis of OS revealed significant differences between the 2 groups (Figure 2A). The median OS was not achieved in either group, but the patients in the R‐maintenance group exhibited better OS than those in the observation group (3‐year overall survival rate 94.5% vs 89.0%, log‐rank test *P‐*value .0240). We further stratified the patients in the R‐maintenance group according to the interval between the end day of R‐induction and the start day of rituximab maintenance. One hundred and eighty‐eight patients received rituximab maintenance within 3 months (<90 days) after the end of R‐induction, and 72 patients started rituximab maintenance within 91st to 180th day after R‐induction. The patients in each group of R‐maintenance still had better OS than those in the observation group (Figure S1). The 3‐year OS was 89.0% in the observation group, 93.9% in patients receiving rituximab maintenance within 90 days, and 95.6% in patients receiving rituximab within 91st to 180th days after R‐induction.

In multivariate Cox proportional hazard models, rituximab maintenance remained significantly associated with a superior OS (adjusted HR 0.42, 95% CI 0.19‐0.92; Table 2). Older age (≥60 years) and stage IV disease also negatively influenced OS in univariate and multivariate analyses (Table 2).

**Table 2:** Univariate and multivariate analyses of overall survival

Variable	Univariate			Multivariate		
	HR	95% CI	*P*‐value	HR	95% CI	*P*‐value
Treatment						
Observation	1.00		.0285^a^	1.00		.0283^a^
R‐maintenance	0.43	(0.20, 0.91)		0.42	(0.19, 0.91)	
Gender						
Female	1.00		.3759	1.00		.5422
Male	0.71	(0.33, 1.52)		0.78	(0.35, 1.73)	
Age (years)						
20‐59	1.00		.0064^a^	1.00		.0252^a^
More than 60	2.97	(1.36, 6.48)		2.58	(1.13, 5.91)	
Charlson comorbidity index						
0	1.00		.1944	1.00		.4384
1	2.01	(0.84, 4.79)		1.65	(0.68, 4.01)	
2+	2.02	(0.72, 5.61)		1.70	(0.59, 4.88)	
Ann Arbor stage						
II‐III	1.00		.0375^a^	1.00		.0258^a^
IV	2.29	(1.05, 5.01)		2.47	(1.12, 5.47)	
Practice setting						
Medical center	1.00		.1620	1.00		.0643
Others	1.75	(0.80, 3.82)		2.21	(0.95, 5.12)	
Induction treatment						
R‐CHOP	1.00		.9926	1.00		.3941
R‐others	1.00	(0.47, 2.17)		0.70	(0.31, 1.59)	
Rituximab cycles in induction treatment						
4‐6 cycles	1.00		.9857	1.00		.6301
7‐8 cycles	1.01	(0.45, 2.24)		0.82	(0.36, 1.86)	

R‐maintenance, rituximab maintenance.

a *P*‐value is <.05.

### 3.3 More patients required further treatments during the first 6 months after the cease of rituximab maintenance

In the Kaplan‐Meier survival analysis and multivariate Cox proportional hazard models of TTF, there was no significant difference between the R‐maintenance and observation groups, and the median TTF was not achieved in both groups (Figure 2B and Table 3). However, when we further closely analyzed the reasons, we found it was closely related to the cease of rituximab maintenance, which was the reimbursement criteria of Taiwan's NHI system.

**Table 3:** Univariate and multivariate analyses of time to treatment failure

Variable	Univariate			Multivariate		
	HR	95% CI	*P*‐value	HR	95% CI	*P*‐value
Treatment						
Observation	1.00		.8463	1.00		.8342
R‐maintenance	1.04	(0.71, 1.51)		0.96	(0.65, 1.42)	
Gender						
Female	1.00		.5929	1.00		.4896
Male	1.10	(0.77, 1.57)		1.13	(0.79, 1.62)	
Age						
20‐59	1.00		.6018	1.00		.8259
60+	1.10	(0.77, 1.59)		1.05	(0.70, 1.56)	
Carlson comorbidity index						
0	1.00		.7463	1.00		.7216
1	0.85	(0.53, 1.36)		0.83	(0.51, 1.34)	
2+	1.06	(0.63, 1.76)		1.01	(0.59, 1.71)	
Ann Arbor stage						
II‐III	1.00		.1597	1.00		.1159
IV	1.29	(0.91, 1.84)		1.34	(0.93, 1.91)	
Practice setting						
Medical center	1.00		.2115	1.00		.1337
Others	0.76	(0.50, 1.17)		0.72	(0.46, 1.11)	
Induction treatment						
R‐CHOP	1.00		.2173	1.00		.1107
R‐others	1.25	(0.88, 1.78)		1.36	(0.93, 1.99)	
Rituximab cycles in induction treatment						
4‐6 cycles	1.00		.0747	1.00		.0357^a^
7‐8 cycles	0.69	(0.46, 1.04)		0.64	(0.42, 0.97)	

R‐maintenance, rituximab maintenance.

a *P*‐value is <.05.

In the end of the first year, patients in the R‐maintenance group exhibited a superior TTF than those in the observation group (1‐year TTF, 89.2% vs 80.7%, respectively; Figure 2B). However, after the eighteenth month (ie, 2‐2.5 years after rituximab maintenance had been initiated, given that the index date here was the 180th day after the initiation of rituximab maintenance), the rate of initiating subsequent treatments dramatically increased in the R‐maintenance group. In the second year after the index date, the TTF of the patients in the R‐maintenance group reduced rapidly (2‐year TTF, 69.0%; 3‐year TTF, 65.1%; Figure 2B). In contrast, the TTF declined stably in the observation group (1‐year TTF, 80.7%; 2‐year TTF, 75.0%; and 3‐year TTF, 68.8%; Figure 2B).

The dramatically reverse of TTF between 2 groups was related to the cease of the 2‐year rituximab maintenance, which was recommended by the reimbursement criteria of Taiwan's NHI system. In the first 6 months after rituximab maintenance was stopped, 22.70% patients need subsequent treatments. The rate was much higher than that during the seventh to twelfth months (4.62%), and that after the thirteenth month (4.62%).

### 3.4 Patients with older age or advanced stages benefited more from rituximab maintenance

**Figure 3:**
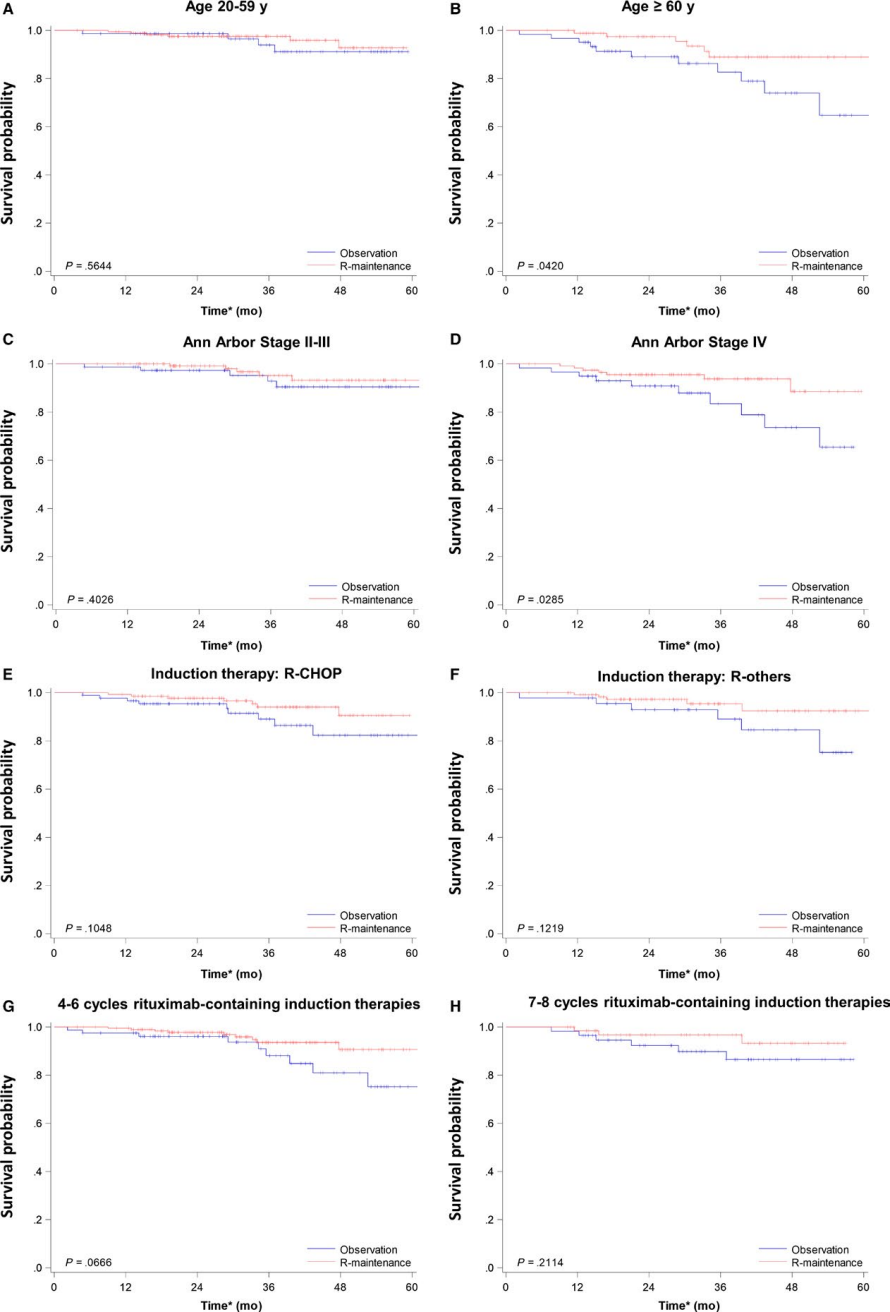
Kaplan‐Meier plots of overall survival in different patient subgroups. A, Patients aged between 20 and 59 y. B, Patients aged older than 60 y. C, Patients with stage II or III FL. D, Patients with stage IV FL. E, Patients receiving R‐CHOP as induction chemotherapy. F, Patients receiving rituximab‐containing induction chemotherapies other than R‐CHOP. G, Patients receiving 4‐6 cycles of rituximab‐containing induction chemotherapies. H, Patients receiving 7 or 8 cycles of rituximab‐containing induction chemotherapies. *The index date (Day 0) was the 180th day after the end date of the last rituximab‐containing induction chemotherapies. R‐others, rituximab‐containing induction chemotherapies other than R‐CHOP

Given that rituximab maintenance improved the OS of patients with FL in Taiwan, we tried to identify a subgroup of patients who would benefit more from rituximab maintenance. We stratified patients based on age, Ann Arbor stages, induction chemotherapies, and cycles of rituximab‐containing induction chemotherapies. Subgroup analyses revealed that patients older than 60 years tended to have better OS when they received rituximab maintenance, compared with those aged between 20 and 59 years (Figure 3A,B). Patients with stage IV disease also benefited from rituximab maintenance compared with those with stage II or III diseases (Figure 3C,D). However, the types or the cycles of induction chemotherapies could not predict the effects of rituximab maintenance (Figure 3E‐H). Therefore, older patients or patients with advanced stages of FL would improve their overall survival when they received rituximab maintenance.

## 4 DISCUSSION

The clinical benefit of rituximab maintenance in FL is equivocal according to previous studies.^16^, ^17^, ^18^, ^19^ Although PFS is improved in patients receiving rituximab maintenance, there is no difference in OS.^16^ On the other hand, limited clinical data are available regarding the clinical use of rituximab maintenance in Asian patients. Our study is the first one to demonstrate the improvement of OS in Asian patients with FL receiving rituximab maintenance in the real‐world setting.

Compared with a previous Japanese phase II study, several differences in our study should be noted.^21^ First, we stratify the patients into 2 groups, the R‐maintenance and the observation groups, to investigate the clinical relevance of rituximab maintenance. The Japanese study is designed as a phase II single‐arm study and is aimed to show the safety of rituximab maintenance in Asian patients with indolent BCL.^17^ Our study further demonstrates the survival benefit of rituximab maintenance in FL and shows that patients with older age or stage IV disease exhibit a survival advantage when they receive rituximab maintenance. Although our study is a retrospective design, it yields more information than the previous one. Second, our cohort is originated from the TCRD and the NHIRD, which include >90% incident cases and their treatments in Taiwan.^22^ The results reflect a nationwide reality of rituximab maintenance in our real‐world practice. Although detailed clinical information is not available, including FLIPI scores, relapse time, and complications of treatments, we still apply several surrogate factors to answer questions. For example, FLIPI scores are not available in our cohort, but we use age, Ann Arbor stage, and CCIs to assess the baseline patient characteristics (Table 1). Given that information regarding refractory and relapse status is not available in our cohort, we use TTF as a surrogate for PFS. Because FL is an indolent lymphoma, most patients experience symptom‐free relapses. These patients do not receive further therapies until they develop disease‐related symptoms. Therefore, several hematologists also suggest that TTF is more feasible and important than PFS in FL.

On the other hand, 2 studies investigate the survival benefits of rituximab maintenance in FL patients.^14^ The first study utilizing data from National LymphoCare Study in the United States prospectively demonstrates that rituximab maintenance improves PFS (HR, 0.68; 95% CI, 0.56‐0.84) and TTF (HR, 0.66; 95% CI, 0.52‐0.84) instead of OS (HR, 0.81; 95% CI, 0.58‐1.14).^19^ The other study retrospectively analyses data from the Czech Lymphoma Study Group (CLSG) database and shows that rituximab maintenance significantly prolongs PFS and OS (5‐year PFS, 74.1% in R‐maintenance vs 52.3% in observation, *P*‐value <.001; 5‐year OS, 93.8% in R‐maintenance vs 87.5% in observation, *P*‐value .005).^15^ The conflicting results might be originated from the differences in patient selections and the definitions of survivals. In contrast to the National LymphoCare Study in the US,^14^ we exclude patients with Ann Arbor stage I and those receiving only R‐monotherapy as induction therapy. Our subgroup analysis illustrates that rituximab maintenance only prolongs OS in patients with advanced‐stage FL (Figure 3D) instead of those with earlier stage FL (Figure 3C). Although we do not enroll patients with stage I FL, we hypothesize that the survival benefit might be diminished when we include more patients with early‐stage diseases. The retrospective Czech study only includes patients receiving R‐CHOP as induction and uses PFS instead of TTF.^15^ In our subgroup analysis, patients receiving R‐CHOP as induction chemotherapies also tend to benefit from rituximab maintenance, but it is not statistically significant (Figure 3E, *P*‐value .1048). This finding might be attributed to lower patient numbers in our cohort. Otherwise, our trend is similar to that of the Czech study. In addition, the results between TTF and PFS might be slightly different. Therefore, further prospective studies are indicated to investigate the Taiwanese population in more detail.

Furthermore, our study raises a question how long is enough for rituximab maintenance. In the observation group, the decline of TTF is stable during the follow‐up (Figure 2B). Although TTF in the R‐maintenance group is better than that in the observation group in the first year, it drops dramatically during the second year, especially during the nineteenth to twenty‐forth month of the follow‐up duration (Figure 2B). Because the index date of our study is the 180th day after R‐induction, the nineteenth to twenty‐forth month is around 2‐2.5 years after R‐induction, which is compatible with the duration of rituximab maintenance approved by the NHI in Taiwan. Meanwhile, majority of the patients in the R‐maintenance group only received rituximab when they require further intravenous therapies after the cease of rituximab maintenance (Table 1). Eventually, the OS in the R‐maintenance group is better than that in the observation group (Figure 2A). Here, it raises a question whether a longer schedule of rituximab maintenance improves the overall survival. To answer this question, Christian Taverna et al^26^ performed a phase III study to compare the long‐term rituximab maintenance with the short‐term one. In their study, the induction therapy is weekly rituximab for 4 weeks, and patients are eligible for stratification if they have at least partial responses at the 13th week.^26^ In the short‐term arm, patient receives 4 administrations of rituximab every 2 months. In the long‐term arm, they receive rituximab infusions every 2 months for maximal 5 years or until disease progression. Although the OS and event‐free survival are not different between 2 arms, patients in the long‐term arm have longer PFS.^26^ At the same time, patient in the long‐term arm also experiences more adverse events than those in the short‐term arm (76% vs 50%, respectively, *P*‐value <.001).^26^ Therefore, the cons and pros of longer rituximab maintenance will be warranted for further investigation.

In conclusion, although our study is a retrospective cohort, it is the first study to illustrate the overall survival benefit for Asian patients with FL. In addition, we further identify that a subgroup of FL patients with older age or stage IV disease experience the greatest clinical improvement through rituximab maintenance.

## CONFLICT OF INTERESTS

Hsiao FY, Chen HM, and Ko BS received a research grant sponsored by Roche Products Ltd. (Taiwan).
