# Supplementary material for: Rituximab maintenance improves overall survival in follicular lymphoma: A retrospective nationwide real‐world analysis from Taiwan Cancer Registry Database
Source: Cancer Med. 2018 Jul 15;7(8):3582–91. doi: 10.1002/cam4.1622 (PMC6089160; doi:10.1002/cam4.1622)
Supplement: Supplementary file 1 [file CAM4-7-3582-s001.pdf]

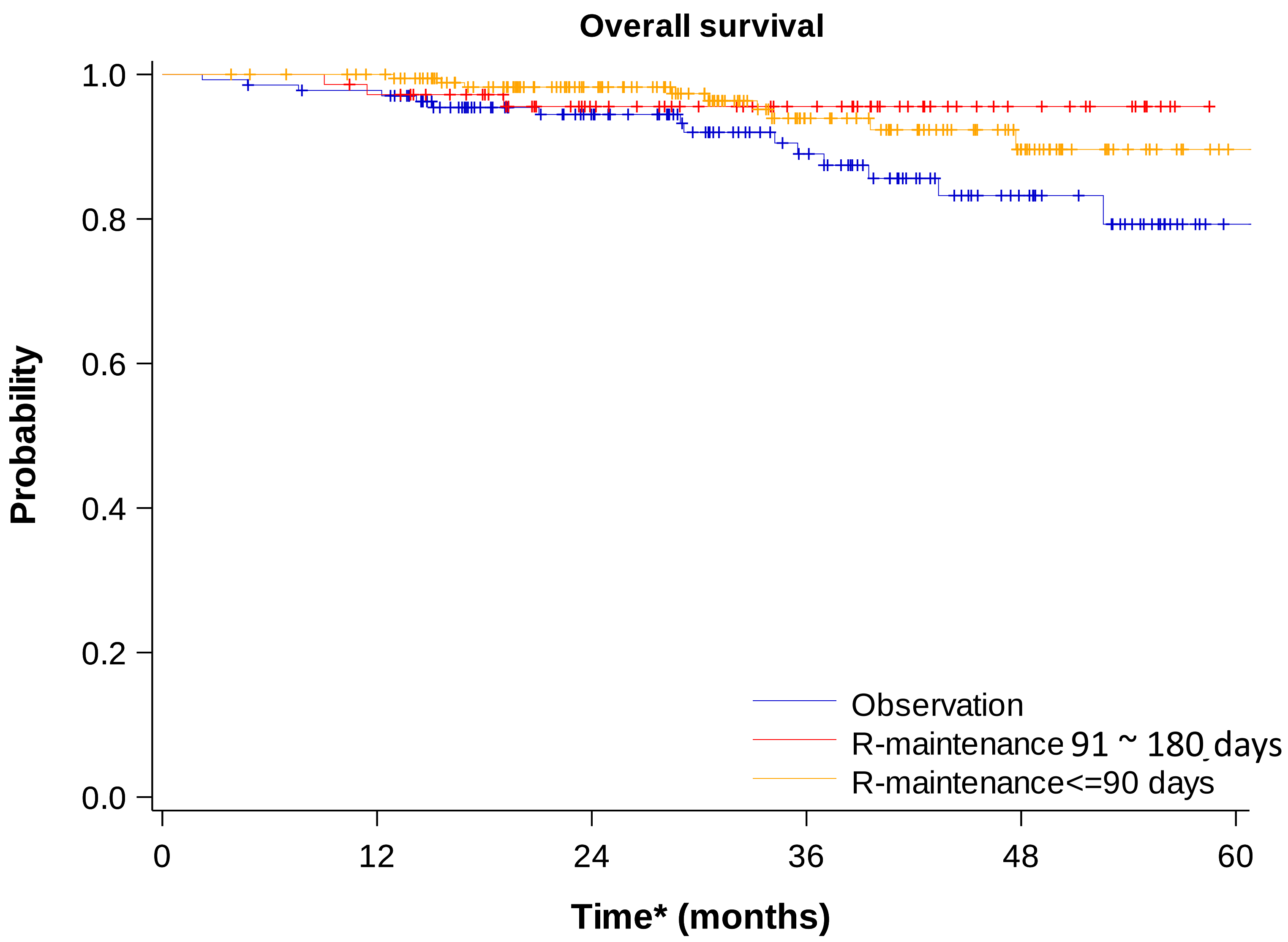

Supplementary Figure 1. Kaplan-Meier Plots of Overall Survival for Enrolled Patients. Patients in the R-maintenance group were stratified according to the start day of rituximab maintenance. Patients in both groups of rituximab maintenance had better overall survival compared with those in the observation group.

* The index date (Day 0) was the 180th day after the end date of the last rituximab-containing induction chemotherapies.
